# Electrophysiological Effects of the Sodium-Glucose Co-Transporter-2 (SGLT2) Inhibitor Dapagliflozin on Human Cardiac Potassium Channels

**DOI:** 10.3390/ijms25115701

**Published:** 2024-05-23

**Authors:** Mara Elena Müller, Finn Petersenn, Juline Hackbarth, Julia Pfeiffer, Heike Gampp, Norbert Frey, Patrick Lugenbiel, Dierk Thomas, Ann-Kathrin Rahm

**Affiliations:** 1 Department of Cardiology, Medical University Hospital Heidelberg, Im Neuenheimer Feld 410, 69120 Heidelberg, Germany; mara.mueller@med.uni-heidelberg.de (M.E.M.); patrick.lugenbiel@med.uni-heidelberg.de (P.L.); dierk.thomas@med.uni-heidelberg.de (D.T.); 2 HCR (Heidelberg Center for Heart Rhythm Disorders), University Hospital Heidelberg, Im Neuenheimer Feld 410, 69120 Heidelberg, Germany; 3 DZHK (German Center for Cardiovascular Research), Partner Site Heidelberg/Mannheim, Heidelberg University, Im Neuenheimer Feld 410, 69120 Heidelberg, Germany

**Keywords:** arrhythmia, dapagliflozin, ion channel, K^+^ channel, repolarization, sodium-glucose co-transporter-2 (SGLT2) inhibitor

## Abstract

The sodium-glucose co-transporter-2 (SGLT2) inhibitor dapagliflozin is increasingly used in the treatment of diabetes and heart failure. Dapagliflozin has been associated with reduced incidence of atrial fibrillation (AF) in clinical trials. We hypothesized that the favorable antiarrhythmic outcome of dapagliflozin use may be caused in part by previously unrecognized effects on atrial repolarizing potassium (K^+^) channels. This study was designed to assess direct pharmacological effects of dapagliflozin on cloned ion channels K_v_11.1, K_v_1.5, K_v_4.3, K_ir_2.1, K_2P_2.1, K_2P_3.1, and K_2P_17.1, contributing to *I*_Kur_, *I*_to_, *I*_Kr_, *I*_K1_, and *I*_K2P_ K^+^ currents. Human channels coded by *KCNH2*, *KCNA5*, *KCND3*, *KCNJ2*, *KCNK2*, *KCNK3*, and *KCNK17* were heterologously expressed in *Xenopus laevis* oocytes, and currents were recorded using the voltage clamp technique. Dapagliflozin (100 µM) reduced K_v_11.1 and K_v_1.5 currents, whereas K_ir_2.1, K_2P_2.1, and K_2P_17.1 currents were enhanced. The drug did not significantly affect peak current amplitudes of K_v_4.3 or K_2P_3.1 K^+^ channels. Biophysical characterization did not reveal significant effects of dapagliflozin on current–voltage relationships of study channels. In conclusion, dapagliflozin exhibits direct functional interactions with human atrial K^+^ channels underlying *I*_Kur_, *I*_Kr_, *I*_K1_, and *I*_K2P_ currents. Substantial activation of K_2P_2.1 and K_2P_17.1 currents could contribute to the beneficial antiarrhythmic outcome associated with the drug. Indirect or chronic effects remain to be investigated in vivo.

## 1. Introduction

Atrial fibrillation (AF) is the most frequent sustained cardiac arrhythmia, with increasing incidence and prevalence over the last few years, and hereby an increasing financial burden on healthcare systems [1,2,3]. Antiarrhythmic management of AF still exhibits suboptimal effectiveness due to a lack of understanding of molecular mechanisms underlying atrial arrhythmogenesis [4,5]. By inhibiting sodium and glucose reabsorption from the proximal tubules, sodium-glucose co-transporter-2 (SGLT2) inhibitors reduce cardiovascular mortality in patients with and without diabetes mellitus. The SGLT2 inhibitor dapagliflozin has been shown to decrease AF incidence in a sub-analysis of the DECLARE-TIMI 58 trial [6] and in a meta-analysis [7]. These antiarrhythmic effects have been attributed in part to antifibrotic and metabolic effects [8]. In addition, direct activating effects of dapagliflozin on voltage-activated potassium currents in vascular tissue [9] or on sodium and inward rectifier potassium currents in human cardiomyocytes derived from induced pluripotent stem cells [10,11] have been reported. Despite the broad use of SGLT2 inhibitors according to current recommendations [12,13], there are aspects with respect to their antiarrhythmic efficacy that remain insufficiently understood. Specifically, potential beneficial antiarrhythmic effects of dapagliflozin on atrial potassium channels have not been systematically investigated to date. We hypothesized that reduced AF rates associated with SGLT2 inhibitor use are in part due to previously unrecognized antiarrhythmic electrophysiological effects of dapagliflozin that are achieved by direct drug effects on atrial repolarizing potassium channels. This study was designed to elucidate the pharmacological effects of dapagliflozin on cardiac ion channels K_v_11.1, K_v_1.5, K_v_4.3, K_ir_2.1, K_2P_2.1, K_2P_3.1 and K_2P_17.1 contributing to human atrial K^+^ currents *I*_Kr_, *I*_Kur_, *I*_to_, *I*_K1_, and *I*_K2P_ that represent potential targets for antiarrhythmic AF therapy [14]. To this end, the respective human ion channel genes *KCNH2*, *KCNA5*, *KCND3*, *KCNJ2*, *KCNK2*, *KCNK3*, and *KCNK17* were heterologously expressed in *Xenopus laevis* oocytes to screen for drug–channel interactions.

## 2. Results

### 2.1. Effects of Dapagliflozin on K_v_11.1 Channels

**Figure 1:**
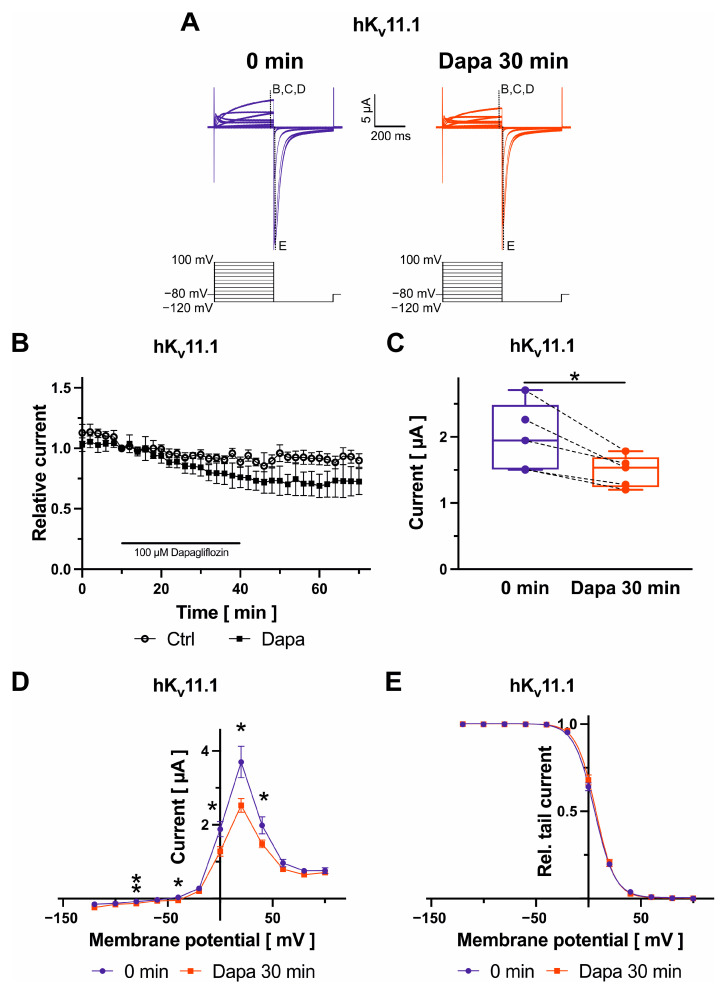
Biophysical effects of dapagliflozin on K_v_11.1 channels. Original current traces evoked with indicated voltage protocols with time-point of analyses marked according to the respective panel (**A**) and maximum current amplitudes at +40 mV are shown before and after administration of 100 µM dapagliflozin ((**B**) Dapa *n* = 5, Ctrl *n* = 11; (**C**) *n* = 5) for 30 min. Currents were normalized to the last current measurement under control conditions (panel (**B**)). Box plots represent median, 25th, and 75th percentiles, as well as minimum and maximum measured value. Dots in panel (**C**) represent raw data. Current–voltage relationships for currents measured during the first step of the voltage protocol are displayed in panel (**D**). Panel (**E**) displays voltage-dependence of activation. To assess voltage-dependence of activation, maximum tail current amplitudes were measured. For each individual oocyte the maximum tail current was set as “1”. Tail currents recorded after test potentials were then normalized to respective maximum tail currents for each cell. No specific normalizing function was applied. Mean values of normalized tail current amplitudes are shown. * *p* < 0.05, ** *p* < 0.01 (please note that *p* values are not indicated in panel (**B**) for clarity of data presentation).

K_v_11.1 channels, underlying the rapidly activating delayed rectifier K^+^ current (*I*_Kr_), were elicited by application of voltage pulses for 400 ms to voltages between −120 mV and +100 mV in 20 mV increments to activate the channels, and tail currents were recorded during a constant repolarizing step to −120 mV for 400 ms (Figure 1A–E). Dapagliflozin was administered at 100 µM for 30 min in all experiments in this study. Activating K_v_11.1 currents were quantified at +40 mV at 390 ms under control conditions and after drug application. Families of current traces from one cell are shown for control conditions and after exposure to dapagliflozin (Figure 1A). After a control period (10 min) with no significant amplitude changes, K_v_11.1 currents decreased upon administration of 100 µM dapagliflozin by 23.8 ± 3.9% (*n* = 5; *p* = 0.020) (Figure 1B,C). Inhibitory effects were virtually irreversible after 30 min following removal of the drug (Figure 1B). In comparison to amplitudes in the presence of dapagliflozin, current levels after drug wash-out did not significantly differ (numerical difference: 4.9 ± 2.9%; *n* = 5; *p* = 0.12). The effects of dapagliflozin on K_v_11.1 current voltage (*I*–*V*) relationship were investigated under isochronal recording conditions. Currents activated at potentials greater than −40 mV, reached a peak at +20 mV and then decreased at more positive potentials due to inactivation (Figure 1D). Figure 1E displays voltage-dependence of activation. Curves were fitted with a single-power Boltzmann distribution of the form *I*_tail_ = *I*_tail.max_/[1 + e^(*V*1/2 − *V*)/*k*^], where *V* is the test pulse potential, *V*_1/2_ is the half-maximal activation potential, and *k* is the slope. The half-maximal activation voltage was not significantly affected by dapagliflozin (5.8 ± 0.9 mV, *k*_control_ = −9.6 ± 0.2; *V*_1/2,dapagliflozin_ = 7.1 ± 1.2 mV, *k*_dapagliflozin_ = −9.1 ± 0.3; *n* = 5; *p_V_*_1/2_ = 0.210).

### 2.2. Effects on K_v_1.5 Channels

**Figure 2:**
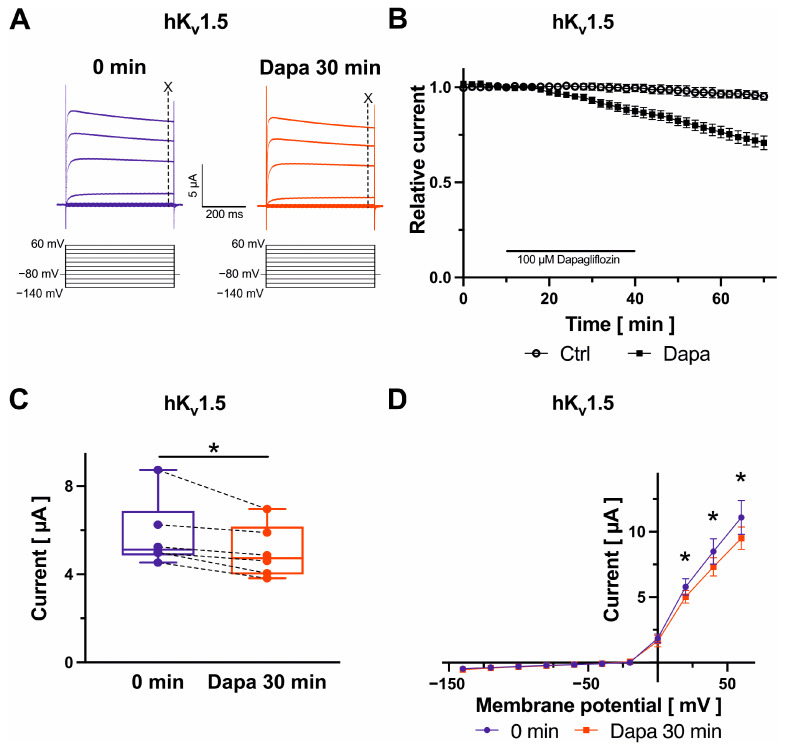
Biophysical effects of dapagliflozin on K_v_1.5 channels. Original current traces evoked with indicated voltage protocols with time-point of analyses marked with dashed lines and “X” (**A**) and maximum current amplitudes at +20 mV are displayed before and after superfusion of cells with 100 µM dapagliflozin ((**B**) Dapa *n* = 6, Ctrl *n* = 4; (**C**) *n* = 6) for 30 min. Currents were normalized to the last current measurement under control conditions (panel (**B**)). Box plots represent median, 25th, and 75th percentiles, as well as minimum and maximum measured value. Dots in panel (**C**) represent raw data. Current–voltage relationships are provided in panel (**D**); * *p* < 0.05 (please note that *p* values are not indicated in panel (**B**) for clarity of data presentation).

K_v_1.5 currents, underlying the ultra-rapid delayed rectifier K^+^ current, were evoked with a pulse step protocol consisting of a variable first voltage step ranging from −140 mV to +60 mV (20 mV increments; 500 ms; Figure 2A). Maximum K_v_1.5 currents measured at +20 mV at 490 ms were reduced after dapagliflozin application (100 µM) by 12.6 ± 2.7% (*n* = 6; *p* = 0.020) (Figure 2B,C). Inhibitory effects further increased by 18.8 ± 4.5% following removal of the drug compared to amplitudes in the presence of dapagliflozin (*n* = 6; *p* = 0.015) (Figure 2B). There were no apparent changes in current–voltage relationships after administration of dapagliflozin (Figure 2D).

### 2.3. Effects on K_v_4.3 Channels

**Figure 3:**
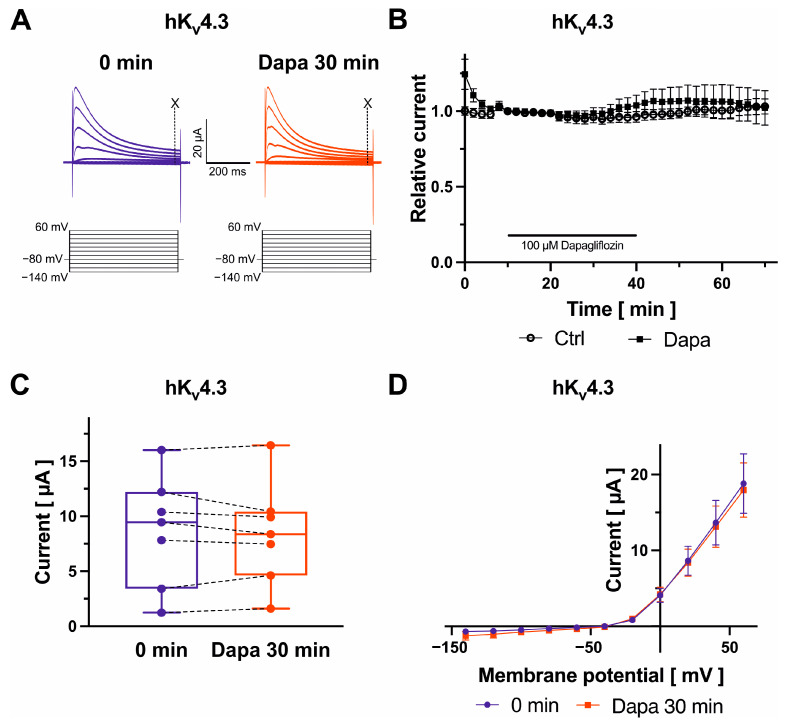
Biophysical effects of dapagliflozin on K_v_4.3 channels. Representative current traces evoked with indicated voltage protocols with time-point of analyses marked with dashed lines and “X” (**A**) and maximum current amplitudes at +20 mV are shown before and after superfusion of cells with 100 µM dapagliflozin ((**B**) Dapa *n* = 7, Ctrl *n* = 5; (**C**) *n* = 7) for 30 min. Currents were normalized to the last current measurement under control conditions (panel (**B**)). Box plots represent median, 25th, and 75th percentiles, as well as minimum and maximum measured value. Dots in panel (**C**) represent raw data. Current–voltage relationships are displayed in panel (**D**).

K_v_4.3 channels, underlying the transient outward K^+^ current, were analyzed using the following protocol: From a holding potential of −80 mV, cells were depolarized to voltages between −140 mV and +60 mV (500 ms, 20 mV increments) (Figure 3A). Peak current amplitudes were recorded and quantified at +20 mV at 490 ms. Dapagliflozin did not induce significant effects on K_v_4.3 peak currents (Figure 3A–D). In addition, analysis of K_v_4.3 outward current–voltage relationships did not indicate substantial modification of biophysical K_v_4.3 characteristics in the presence of dapagliflozin (Figure 3D).

### 2.4. Effects on K_ir_2.1 Channels

**Figure 4:**
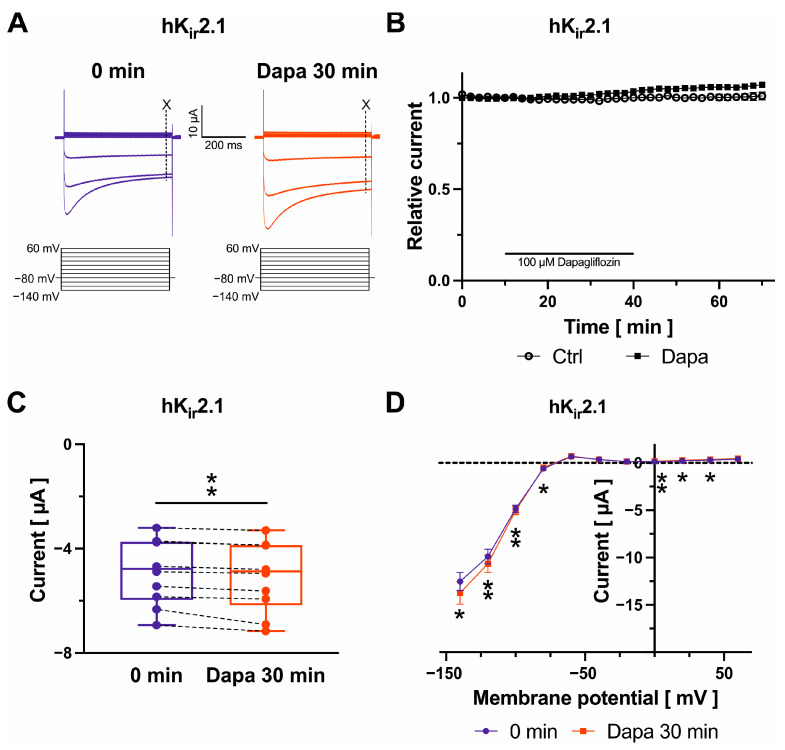
Biophysical effects of dapagliflozin on K_ir_2.1 channels. Typical current traces evoked with indicated voltage protocols with time-point of analyses marked with dashed lines and “X” (**A**) and maximum current amplitudes at −100 mV are shown before and after superfusion of cells with 100 µM dapagliflozin ((**B**) Dapa *n* = 10, Ctrl *n* = 5; (**C**) *n* = 10) for 30 min. Currents were normalized to the last current measurement under control conditions (panel (**B**)). Box plots represent median, 25th, and 75th percentiles, as well as minimum and maximum measured value. Dots in panel (**C**) represent raw data. Current–voltage relationships are displayed in panel (**D**); * *p* < 0.05, ** *p* < 0.01 (please note that *p* values are not indicated in panel (**B**) for clarity of data presentation).

K_ir_2.1 inward rectifier currents were recorded during test pulses from −140 mV to +60 mV in 20 mV increments (500 ms) (Figure 4A). Current amplitudes at –100 mV were determined to quantify dapagliflozin effects at 490 ms. Typical recordings under control conditions following a current equilibration period and in the presence of dapagliflozin are displayed in Figure 4A. K_ir_2.1 (Figure 4B,C) inward current amplitudes exhibited a minor increase after drug application by 3.5 ± 0.7% (*n* = 10; *p* = 0.005) compared with drug-free control measurements. Current amplitudes continued to increase slightly during 30 min after removal of the drug compared to amplitudes in the presence of dapagliflozin (+3.6 ± 0.7%; *n* = 10; *p* = 0.002) (Figure 4B). K_ir_2.1 current-activation kinetics were not altered by dapagliflozin (Figure 4D).

### 2.5. Dapagliflozin Effects on K_2P_2.1, K_2P_3.1, and K_2P_17.1 Channels

**Figure 5:**
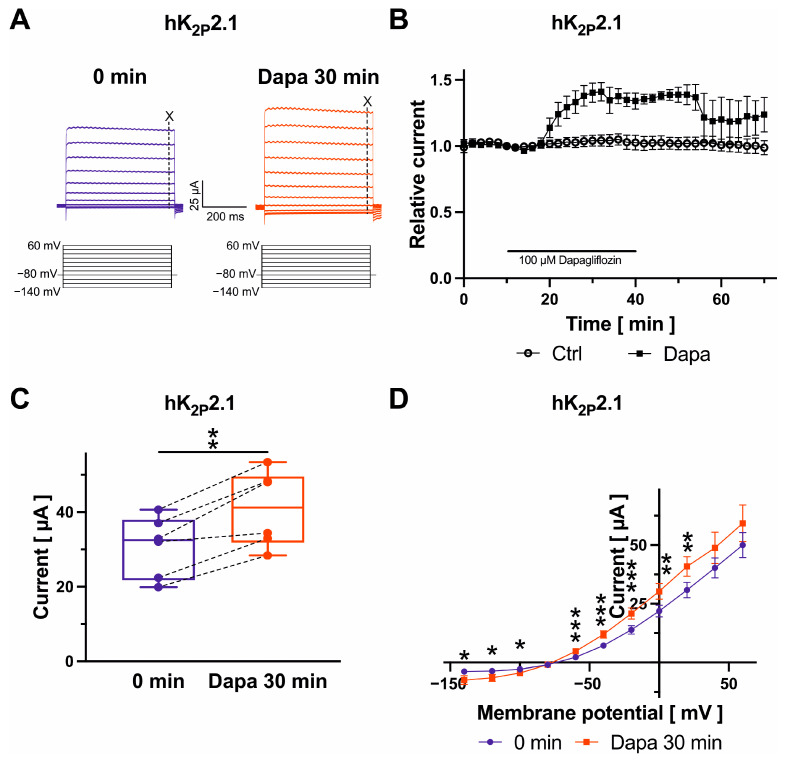
Biophysical effects of dapagliflozin on K_2P_2.1 channels. Representative recordings evoked with indicated voltage protocols with time-point of analyses marked with dashed lines and “X“ (**A**) and maximum current amplitudes at +20 mV are provided before and after superfusion of cells with 100 µM dapagliflozin ((**B**) Dapa *n* = 6, Ctrl *n* = 7; (**C**) *n* = 6) for 30 min. Currents were normalized to the last current measurement under control conditions (panel (**B**)). Box plots represent median, 25th, and 75th percentiles, as well as minimum and maximum measured value. Dots in panel (**C**) represent raw data. Current–voltage relationships are displayed in panel (**D**); * *p* < 0.05, ** *p* < 0.01, *** *p* < 0.001 (please note that *p* values are not indicated in panel (**B**) for clarity of data presentation).

**Figure 6:**
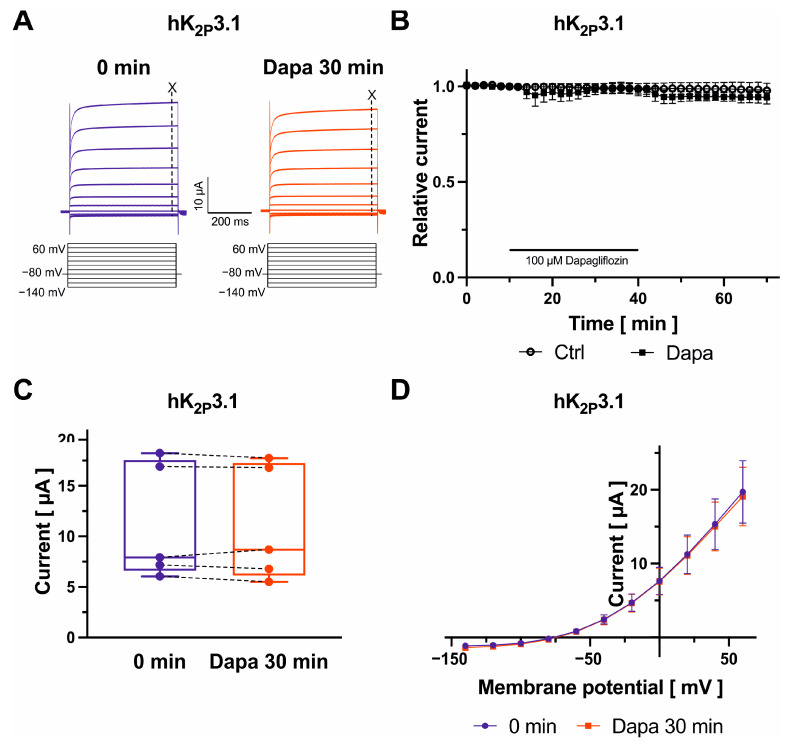
Biophysical effects of dapagliflozin on K_2P_3.1 channels. Representative recordings evoked with indicated voltage protocols with time-point of analyses marked with dashed lines and “X” (**A**) and maximum current amplitudes at +20 mV are provided before and after superfusion of cells with 100 µM dapagliflozin ((**B**) Dapa *n* = 5, Ctrl *n* = 5; (**C**) *n* = 5) for 30 min. Currents were normalized to the last current measurement under control conditions (panel (**B**)). Box plots represent median, 25th, and 75th percentiles, as well as minimum and maximum measured value. Dots in panel (**C**) represent raw data. Current–voltage relationships are displayed in panel (**D**).

**Figure 7:**
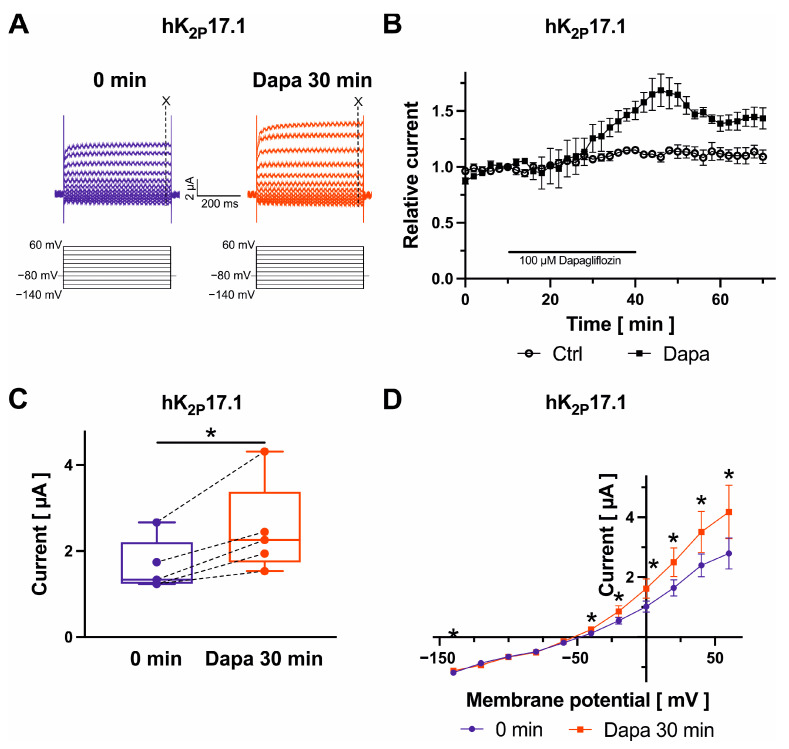
Biophysical effects of dapagliflozin on K_2P_17.1 channels. Representative recordings evoked with indicated voltage protocols with time-point of analyses marked with dashed lines and “X” (**A**) and maximum current amplitudes at +20 mV are provided before and after superfusion of cells with 100 µM dapagliflozin ((**B**) Dapa *n* = 5, Ctrl *n* = 6; (**C**) *n* = 5) for 30 min. Currents were normalized to the last current measurement under control conditions (panel (**B**)). Box plots represent median, 25th, and 75th percentiles, as well as minimum and maximum measured value. Dots in panel (**C**) represent raw data. Current–voltage relationships are displayed in panel (**D**); * *p* < 0.05 (please note that *p* values are not indicated in panel (**B**) for clarity of data presentation).

Finally, K^+^ currents were recorded from oocytes expressing K_2P_ channel subunits 2.1, 3.1, or 17.1, which are contributing to the background K^+^ current carried out by two-pore-domain potassium channels, during test pulses from −140 mV to +60 mV in 20 mV increments (500 ms) (Figure 5A, Figure 6A and Figure 7A). K_2P_ current amplitudes were measured at the end of the +20 mV-pulse at 490 ms. K_2P_2.1 (+34.1 ± 6.1%; *n* = 6; *p* = 0.003) and K_2P_17.1 currents (+50.1 ± 8.2%; *n* = 5; *p* = 0.019) were activated by 100 µM dapagliflozin, whereas K_2P_3.1 current amplitudes were not significantly changed (Figure 5B,C, Figure 6B,C and Figure 7B,C), respectively. Activation of K_2P_2.1 current was not significantly reversible upon drug wash-out for 30 min. Current levels decreased numerically by 5.5 ± 12.3% (*n* = 6; *p* = 0.87) compared to K_2P_2.1 amplitudes in the presence of dapagliflozin (Figure 5B). In addition, activating effects on K_2P_17.1 were virtually irreversible after 30 min following removal of the drug in comparison to amplitudes measured at the end of dapagliflozin application (−3.9 ± 6.6%; *n* = 5; *p* = 0.46) (Figure 7B). K_2P_ currents display outward (or open) rectification that is characteristic to a potassium-selective background leak conductance (Figure 5D, Figure 6D and Figure 7D). There were no apparent dapagliflozin effects on K_2P_2.1, K_2P_3.1, or K_2P_17.1 current rectification or *I/V* curves. The currents showed outward rectification before and after administration of dapagliflozin (Figure 5D, Figure 6D and Figure 7D).

## 3. Discussion

This study provides the first assessment of direct dapagliflozin effects on cardiac potassium channels. The work reveals that human cardiac K_v_1.5, K_v_11.1, K_ir_2.1, K_2P_2.1, and K_2P_17.1 currents are previously unrecognized targets for dapagliflozin. The drug did not significantly affect peak current amplitudes of K_v_4.3 or K_2P_3.1 K^+^ channels. These findings significantly extend the pharmacological profiling of dapagliflozin.

The assessment provided in this study was focused on K^+^ channels underlying human atrial *I*_Kr_, *I*_Kur_, *I*_to_, *I*_K1_, and *I*_K2P_ as potential antiarrhythmic targets of dapagliflozin [14]. We observed substantial activation of human cardiac K_2P_2.1 and K_2P_17.1 channels by ~34% and ~50%, respectively. By contrast, lower degrees of K_v_11.1 and K_v_1.5 block (by ~24% and ~13%, respectively) and of K_ir_2.1 activation (by ~4%) indicate minor relevance of these targets. K_2P_2.1 channels are inhibited by most compounds tested, including antiarrhythmic drugs [15] and they are expressed in the human (and porcine) heart [16,17]. Atrial K_2P_2.1 downregulation has previously been observed in heart failure (HF) patients with AF and in a pig model of AF and HF [16,17], and enhancement of atrial K_2P_2.1 channel expression provides rhythm control in pigs [17]. K_2P_17.1 channels are expressed predominantly in the atria [18]. As opposed to the commonly observed inhibition of cardiac K^+^ channels by drugs, activation of K_2P_17.1 channels has been previously reported upon exposure to antiarrhythmic drugs metoprolol, mexiletine, propafenone, propranolol, quinidine, and vernakalant [19,20]. Atrial K_2P_17.1 abundance is reduced in patients with HF or AF [21], and duration of atrial action potentials (AP) is prolonged in patients and animal models with HF [17,21], consistent with reduction of repolarizing K^+^ currents. Changes in K_2P_17.1 expression and function regulate action potential duration (APD) of cardiac myocytes. Expression of the K_2P_17.1 G88R gain-of-function mutant results in APD shortening in HL-1 murine atrial myocytes [22], while siRNA-mediated knockdown of K_2P_17.1 current causes APD prolongation in cardiomyocytes derived from human induced pluripotent stem cells [23]. Finally, enhancement of repolarizing atrial K^+^ channels observed here is consistent with shortening of APD reported in porcine atrial myocytes upon exposure to dapagliflozin [11]. Thus, in contrast to the “classical” mechanism of atrial arrhythmogenesis in AF involving reduced electrical conduction velocity and shortening of atrial APD and effective refractory periods (AERP) that perpetuate AF through promotion of electrical re-entry [24], we suggest that in HF patients treated with dapagliflozin, pharmacologic activation of K_2P_2.1 and K_2P_17.1 current levels may specifically counteract atrial remodeling and suppress atrial arrhythmias.

Block of the K_v_11.1 channel and hereby the *I*_Kr_-current, is a known cause of APD and consequently QT-time prolongation, which can lead to the occurrence of life-threatening arrhythmias such as torsade-de-pointes-tachycardia [25]. In our study, we were able to show a block of the *I*_Kr_ current by dapagliflozin (100 µM), suggesting an impact on cardiac repolarization. However, no significant prolongation of the QT interval could be demonstrated by dapagliflozin in clinical studies [26,27]. On the contrary, Nakase et al. demonstrated a shortening of the QT interval in patients with heart failure with reduced ejection fraction receiving dapagliflozin therapy [27]. The apparent discrepancy in the potency measured here and observed in clinical studies is most likely due to the increased dosage requirement in oocyte experiments and indicates the importance of the interaction of all repolarizing potassium currents and the overall effects of dapagliflozin. Further, we were also able to show a block of the *I*_Kur_-current by 100 µM dapagliflozin incubation. The K_v_1.5 channel plays an important role in human cardiac repolarization on atrial level [28,29]. A decrease in expression levels of K_v_1.5 protein in human atria in patients with chronic atrial fibrillation has been observed previously [30] and a *KCN5A* loss-of-function mutation has previously been linked to the occurrence of atrial fibrillation [31]. Due to the small blocking effects at high dapagliflozin concentrations we do not expect major clinical impact even though it has already been shown that dapagliflozin reduces the incidence of atrial fibrillation [6].

Taking all data together, we assume a direct interaction between dapagliflozin and the repolarizing potassium channels due to the long-term block, and lack of reversibility of the effects probably due to irreversible drug–channel interaction. However, further investigations are necessary to assess the actual mechanism of channel modulation and the resulting clinical impact.

Drug concentrations applied in this study to reveal electropharmacological actions were relatively high, because drug effects in oocytes generally require 3–10 times higher concentrations compared to mammalian or human cells due to specific properties of oocytes (e.g., the vitelline membrane and the yolk) that reduce the actual concentration of drugs at the cell surface [19]. During therapeutic application of dapagliflozin (10–25 mg o.d.), peak concentrations of 0.4–0.7 µM were detected in patients [32]. Dapagliflozin is approximately 91% bound to plasma proteins [32], reducing effective free drug concentrations and indicating low clinical impact of dapagliflozin on atrial electrophysiology with the current dosing recommendations under regular pharmacokinetic situations. Therefore, associations between in vitro modulation of K_2P_2.1 and K_2P_17.1 and direct clinical effects on these channels cannot be established at the present stage of dapagliflozin electropharmacology research. However, impaired metabolism may result in significantly higher plasma levels and in modulation of K_2P_2.1 and K_2P_17.1 channel function during drug administration.

### Limitations

The primary goal of this study was to specifically investigate direct acute dapagliflozin effects on a candidate panel of human atrial K^+^ channels. Indirect effects of dapagliflozin were beyond the scope of this work. This focus carries inherent limitations: conclusions regarding cardiac ion channels not included in the test panel are not feasible, and chronic effects were not investigated. To provide a complete representation of electrophysiological effects of dapagliflozin further studies are required, with particular focus on additional ion channels and on chronic dapagliflozin effects, as well as dosage and time-dependent effects. Moreover, further studies are needed to assess whether other SGLT-2 inhibitors have stronger, similar, opposite or no effects on cardiac repolarizing potassium channels and whether a clinical consequence can arise from comparison between drugs. Additionally, although oocytes represent a widespread model system for the analysis of pharmacological effects on ion channels, it requires further investigations on human cell systems to determine if there is a direct consequence for everyday clinical practice.

## 4. Materials and Methods

### 4.1. Molecular Biology

Complementary (c)DNA encoding human K_v_11.1 cDNA was supplied by Mark T. Keating (Boston, MA, USA), hK_2P_2.1 was provided by Steve Goldstein (Irvine, CA, USA), hK_v_1.5 and hK_v_4.3 clones were provided by Barbara A. Wible (Cleveland, OH, USA), and hK_ir_2.1 DNA was donated by Carol A. Vandenberg (Santa Barbara, CA, USA). Human K_2P_3.1 and hK_2P_17.1 were cloned as described previously [33,34]. For in vitro transcription, cDNAs were subcloned into expression vectors containing a T7 promoter for cRNA synthesis. Plasmids were linearized and transcribed using the T7 mMessage mMachine kit (Ambion, Austin, TX, USA). RNA transcripts were quantified by spectrophotometry (NanoDrop 2000, Thermo Fisher Scientific, Waltham, MA, USA) and subjected to separation by agarose gel electrophoresis to assess RNA integrity.

### 4.2. Xenopus Laevis Oocyte Preparation

Ovarian lobes were surgically removed in aseptic technique from female *Xenopus laevis* frogs anesthetized with 1 g/L tricaine solution (pH = 7.5). Frogs were not fed on the day of surgery to avoid emesis during anesthesia. After surgery, the frogs were allowed to recover consciousness, followed by at least two months recovery period. Oocyte collection was alternated between left and right ovaries, and no more than three surgeries were performed on one individual frog. After the final taking of oocytes, the anesthetized frog was killed by decerebration and pithing. Following collagenase treatment (collagenase D; Roche Diagnostics, Mannheim, Germany), stage V–VI defolliculated oocytes were manually sorted under a stereomicroscope. For electrophysiological recordings, complementary (c)RNA (1.5–25 ng; 46 nL/oocyte) encoding study channels was injected.

### 4.3. Electrophysiology

Two-electrode voltage clamp measurements were performed to record whole-cell currents from *Xenopus laevis* oocytes 2 days after cRNA injection. Two-electrode voltage clamp electrodes were pulled from 1 mm borosilicate glass tubes (GB100F-10, Science Products, Hofheim, Germany) using a P-1000 micropipette puller (Sutter Instruments, Novato, CA, USA). Macroscopic currents were recorded using an Oocyte Clamp amplifier OC-725C (Warner Instruments, Hamden, CT, USA) and pClamp 8.2 software (Molecular Devices, Sunnyvale, CA, USA). Data were sampled at 2 kHz and low-pass filtered at 1 kHz. Peak currents were measured relative to zero current level. Leak currents were not subtracted. Voltage clamp measurements were carried out at room temperature (20–22 °C). Voltage clamp electrodes filled with a 3 mM K^+^ solution had tip resistances of 5–15 MΩ. Experiments were performed under constant perfusion by a gravity-driven perfusion system. The standard extracellular bath solution contained 101 mM NaCl, 4 mM KCl, 1.5 mM CaCl_2_, 2 mM MgCl_2_, and 10 mM 4-(2-hydroxyethyl)-1-piperazineethanesulfonic acid (HEPES). The pH was adjusted with NaOH to pH 7.4, except for K_2P_17.1 (pH 8.5). The holding potential was −80 mV in all oocyte experiments performed in this study. All measurements included a 10-min equilibration phase, followed by a 30-min wash-in period with 100 µM dapagliflozin to create a sufficient exposure time to reach a steady-state for deriving and analyzing possible effects, and by a 30-min wash-out phase with the bath solution in order to check for reversibility of identified effects. Control experiments were performed under constant perfusion with a standard bath solution. All experiments were performed by applying the indicated voltage protocol in 2-min intervals, respectively. The point of measurement is graphically illustrated in the exemplary current traces.

### 4.4. Drugs

Dapagliflozin was obtained from Sigma Aldrich (St. Louis, MO, USA), dissolved in dimethyl sulfoxide (DMSO) to a 100 mM stock solution, and stored at −20 °C. Aliquots of the stock solutions were diluted to the desired concentration with the bath solution on the day of experiments. The solvent (0.1% DMSO; 30 min) did not significantly affect mean current amplitudes of study channels.

### 4.5. Data Analysis and Statistics

PClamp 8.2 (Axon Instruments, Foster City, CA, USA), Origin 2023 (OriginLab, Northampton, MA, USA), Excel version 16.85 (Microsoft, Redmond, WA, USA), CorelDRAW Graphics Suite 2023 (Corel, Ottawa, Canada), and Prism 8.0 (GraphPad, La Jolla, CA, USA) software were used for data acquisition, analysis, and presentation. Data are expressed as mean ± standard error of the mean (SEM). Paired and unpaired Student’s *t*-tests (two-tailed tests) were applied to compare statistical significance of the results where appropriate. *p* < 0.05 was considered as statistically significant. The Kolmogorov–Smirnov test was used to test data sets for normal distribution.

## 5. Conclusions

Human atrial potassium channels are differentially regulated by dapagliflozin. K_2P_2.1 and K_2P_17.1 current increase by dapagliflozin represents a previously unrecognized mode of electropharmacological action of an SGLT2 inhibitor. This work highlights a mechanistic link between activation of cardiac K^+^ currents and clinical antiarrhythmic effects. K^+^ channel activation by dapagliflozin may be employed for personalized rhythm control in HF patients exhibiting reduction of atrial K^+^ channel function. The therapeutic significance of K^+^ channel modulation by dapagliflozin requires validation in translational and clinical studies.

## Data Availability

The authors confirm that the data supporting the findings of this study are available within the article.
